# Intrapelvic Kirschner wire Migration following Developmental Dysplasia of Hip Surgery and Laparoscopic Removal

**DOI:** 10.12669/pjms.39.6.7413

**Published:** 2023

**Authors:** Muhammad Abdul Basit, Ahmed Shams, Shahzad Anver Qureshi, Mumtaz Hussain

**Affiliations:** 1 Muhammad Abdul Basit, Senior Registrar of Paediatric Orthopaedics, The Children’s Hospital, Faisalabad, Pakistan; 2 Ahmed Shams, Clinical Fellow of Paediatric Orthopaedic Surgery, The Children’s Hospital & University of Child Health Sciences, Lahore, Pakistan; 3 Shahzad Anver Qureshi, Senior Registrar of Paediatric Orthopaedic Surgery The Children’s Hospital & University of Child Health Sciences, Lahore, Pakistan; 4 Mumtaz Hussain, HOD & Assistant Professor of Paediatric Orthopaedic Surgery, The Children’s Hospital & University of Child Health Sciences, Lahore, Pakistan

**Keywords:** Femoral shortening, Salter innominate osteotomy, Paediatric Orthopaedics

## Abstract

Salter innominate osteotomy remains the most commonly performed pelvic osteotomy for the Developmental Dysplasia of Hip in children after 18 months of age up to six years. Kirschner wire (K- wire) is used to fix the bone graft across the osteotomy site. Of the several complications of the pelvic osteotomy, K- wire migration into the pelvis is rare and only a few case reports are reported. We present a case of a 2-year-old girl with Right sided Developmental Dysplasia of Hip who underwent Femoral shortening and Salter innominate osteotomy, presented three months later with intrapelvic migration of k-wire. Paediatric Surgery consult was obtained and K-wire was removed laparoscopically without any complications successfully.

## INTRODUCTION

Developmental Dysplasia of Hip (DDH) is a condition of abnormal development with dysplastic features of the femoral head and acetabulum often leading to subluxation or dislocation of the affected hip joint. Depending on the age at presentation and the underlying features, management ranges from Pavlik Harness to Open Reduction along with pelvic osteotomies. Of the variety of pelvic osteotomies performed, Salter Innominate Osteotomy (SIO) is the most commonly performed in the surgical management of DDH.^1^ Autogenous cortico-cancellous Iliac graft is taken and placed following osteotomy between the proximal iliac and distal acetabular fragments which is then secured with two k-wires. Where k-wires are used, migration is a rare complication such as in proximal humerus,^2^ but only a few cases of intrapelvic migration have been reported so far. In this case study we report a case of SIO for DDH in a 2-year-old girl, with pelvic migration of k-wire which was successfully removed without any damage to the internal viscera.

## CASE REPORT

**Fig.1:**
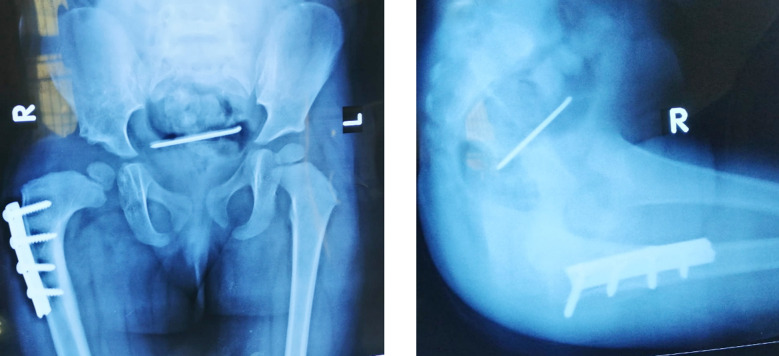
Plain Radiograph Anteroposterior and Lateral Projection of pelvis showing the location of intrapelvic location of k-wire.

A 2-year-old girl presented to the outpatient department of Children Hospital and University of Child Health Sciences after being referred from a Private Hospital, eight weeks earlier she had undergone SIO along with Femoral shortening for right sided DDH. There was no active complaint at presentation. Of the two k-wires, one had been removed and the other wire seemed to have migrated into the pelvis (Fig.1) for which she had been referred to the tertiary care Hospital. When thoroughly explained to the parents regarding this complication and the need for surgical intervention for the removal of wire, parents were reluctant to have their child undergo any surgery at first, but detailed counselling by both the Orthopedic and the surgical team regarding the possible adverse effects and minimal invasive laparoscopic approach, the parents gave consent for surgery at 3^rd^ month after index surgery. Despite the delay in removal of k-wire, the patient remained well and did not develop any clinical signs of intrapelvic organ injury. Plan for removal of k-wire was formulated. Following medical evaluation, laparoscopic removal of k-wire by the pediatric surgical team and removal of the femoral plate by the Orthopedic team was successfully performed. The k-wire was found above the urinary bladder and uterus. No injury to other organs was seen per-operatively. The child recovered uneventfully.

**Fig.2:**
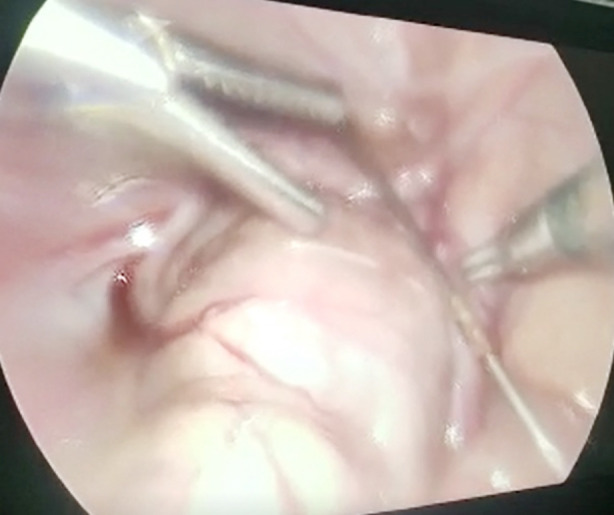
Intra-Pelvis location of K-wire, Laparoscopic view.

**Fig.3:**
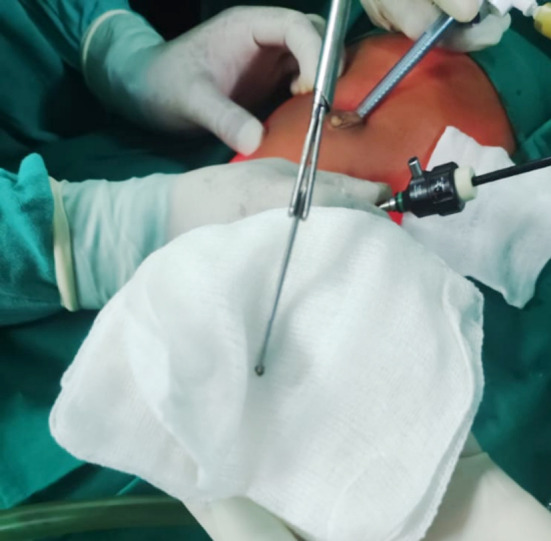
The Retrieved K-wire.

## DISCUSSION

No procedure is free of complications and SIO is no exception. Most common complication being Avascular Necrosis of the femoral head,^3^ K-wire migration is relatively rare with most case reports showing intrathoracic migration of k-wire following fixation of proximal humerus fractures.^4^ Pin migration outside the abdomen or pelvis following SIO has been well studied and reported.^5^ Although prevalence of intra-abdominal or intra-pelvic pin migration is unknown, it is a well-known complication and is believed to be increasing with most cases being underreported. One study in 2008 revealed intraabdominal migration of k-wire following SIO in a 4-year old girl which was also removed via mini-incision.^6^ There have been studies showing injury to intraabdominal and intra-pelvic organs such as urinary bladder following k-wire migration,^7^ which can lead to serious manifestations.

Multiple reasons have been thought to cause k-wire migration including gravity, smooth un-bent k-wire capillary action and muscle contraction.^4^,^8^ In our case the most likely reason seemed to be inadequacy of bend at proximal end of the smooth k-wire used. However, in literature, all kind of pins such as threaded or bent have been reported to migrate.^2^,^8^

To prevent this potentially fatal complication we recommend to use threaded pin and to bend the proximal end of the k-wire, using threaded of locked pins, usage of restraining devices and to withdraw the k-wires at the end of treatment, and with close clinical as well as radiological follow-up. Recent advances may include the use of bioabsorbable rods in children less than five years of age.^9^ Some experts have also recommended removal of k-wires at the time of removal of cast.^10^

In summary we recommend to bend the proximal end of k-wires and to use threaded pins, and in case of identified pin migration, it should be removed immediately to prevent catastrophic complications in children. Pediatric Surgical team should also be consulted to formulate the best plan for the removal of k-wire.

### Authors’ Contribution:

**MAB:** Conceived the Idea, Designed Research, Literature Review, Case collection.

**AS:** Literature Search, Manuscript Writing.

**SAQ:** Statistical Analysis.

**MH:** Manuscript final reading and approval.
